# Transcriptomic Analysis of Postnatal Rat Carotid Body Development

**DOI:** 10.3390/genes15030302

**Published:** 2024-02-27

**Authors:** Ning Wang, Ying-Jie Peng, Wenjun Kang, Matthew Hildreth, Nanduri R. Prabhakar, Jayasri Nanduri

**Affiliations:** 1 Institute for Integrative Physiology and Center for Systems Biology of O_2_ Sensing, The University of Chicago, Chicago, IL 60637, USA; 2 Center for Research Informatics, The University of Chicago, Chicago, IL 60637, USA

**Keywords:** carotid body, hypoxia, RNA-Seq, oxidative phosphorylation, mitochondria

## Abstract

The carotid body (CB), located bilaterally at the carotid artery bifurcations, is the primary sensory organ for monitoring arterial blood O_2_ levels. Carotid bodies are immature at birth, exhibiting low sensitivity to hypoxia, and become more sensitive with maturation during the first few weeks of neonatal life. To understand the molecular basis for the postnatal developmental hypoxic responses of CB, we isolated CBs from 5-day and 21-day-old Sprague–Dawley rats and performed RNA sequencing, which allows comprehensive analysis of gene expression. Differentially expressed genes (DEGs) were generated using Edge R, while functional enrichment analysis was performed using gene-set enrichment analysis (GSEA). Analysis of RNA-Seq data showed 2604 DEGs of the total 12,696 genes shared between neonates and adults. Of the 2604 DEGs, 924 genes were upregulated, and 1680 genes were downregulated. Further analysis showed that genes related to oxidative phosphorylation (Ox/phos) and hypoxia-signaling pathways were significantly upregulated in neonatal CBs compared to adult CBs, suggesting a possible link to differential developmental hypoxic responses seen in CB. Genes related to cytokine signaling (INFγ and TNFα) and transcription factors (CREB and NFΚB) mediated pathways were enriched in adult CBs, suggesting that expression of these pathways may be linked to developmental regulation. The RNA-Seq results were verified by analyzing mRNA changes in selected genes by qRT-PCR. Our results of enrichment analysis of biological pathways offer valuable insight into CB hypoxic sensing responses related to the development process.

## 1. Introduction

Systemic hypoxia, arising due to moderate drops in oxygen (O_2_) levels in the arterial blood, is a fundamental physiological stimulus that can be acute, ranging from seconds to minutes, or chronic, lasting several hours to days. Acute hypoxia at the systemic level evokes rapid changes in the cardiorespiratory systems, which are crucial to ensure optimal O_2_ delivery to tissues and to prevent further widespread hypoxemia [1]. Cardiorespiratory responses to acute hypoxia are initiated by the activation of carotid bodies (CB), which are the major sensory organs for monitoring arterial blood O_2_ levels [2,3]. CB is a highly vascularized tissue and is made of two major cell types: glomus cells (type I), which are of neuronal origin, and sustentacular cells (type II), which resemble glial cells of the nervous system, in addition to minor cell types including endothelial, immune and connective tissue cells [2].

In vitro CB sensory activity recordings show that CBs exhibit low sensitivity to hypoxia at birth and become more sensitive during the first 15–30 days following birth [4,5]. Cellular studies on glomus cells, the primary site of O_2_ sensing, showed that the postnatal increase in CB hypoxic sensitivity is linked to glomus cell maturation, with multiple players being critical to this process [4,6]. Increased density of O_2_-sensitive K+ channels [4,7], increased intracellular calcium levels [8], modulation of mitochondrial metabolism [9], and maturation of neurotransmitters [10] have been reported as possible cellular mechanisms for increased CB response to hypoxia in adults. Another consensus view is that CB O_2_ sensitivity is adapted to the low PaO_2_ environment of the fetus (23–27 mmHg) due to the expression of hERG-like K+ currents [11] and their postnatal downregulation due to the rise in PO_2_ after birth [12,13]. However, a systematic study of genetic factors accounting for developmental changes leading to postnatal increase in CB O_2_ sensitivity has not been addressed.

Next-generation sequencing technologies have revolutionized genomic research to explore transcriptome expression changes without prior assumptions. RNA-seq is a powerful quantitative tool to analyze genome-wide expression, which is closely related to gene functionality. The aim of this study is to use the RNA-Seq dataset to identify the variations in the transcriptome of neonatal pups and adult rats to provide insights into the complex molecular pathways that may play a role in the CB hypoxic sensitivity with development. Although transcriptomic profiling of CB at the whole tissue [14,15,16] and single glomus cell [17] has been reported previously, this represents the first study to comprehensively catalog transcriptome profiles of CBs in two developmental stages (neonate vs. adult), which provides a useful resource to address other aspects related to diseases, drug efficacy, and toxicity mechanisms.

## 2. Materials and Methods

### 2.1. Animal Study

All experimental protocols were approved by the Institutional Animal Care and Use Committee (IACUC) of the University of Chicago (approved protocol #71811). Sprague–Dawley pregnant rats purchased from Charles River Laboratories were used for the experiments. Mother was housed on a 12 h light–dark cycle and fed ad libitum a standard pelleted rodent diet with free access to the water. Pups aged 5 days were sacrificed by IP urethane injection, and bifurcations were harvested. One third of the litter was allowed to grow for 21 days, after which they were sacrificed, and CB bifurcations were harvested. CBs were isolated from the bifurcations and immediately transferred to an RNA stabilizing solution (RNAlater) at 4 °C.

### 2.2. RNA Extraction, Illumine Library Preparation, and Sequencing

Total RNAs were extracted using the Direct-ZOL RNA micro prep kit (Zymo Research; #R2060, Irvine, CA, USA) according to the manufacturer’s instructions. For each experiment, CBs from eight pups (postnatal day 5) or four adult rats (postnatal day 21) were pooled. The experiment was run in 3 biological triplicates, resulting in a total number of 24 P5 pups and 12 P21 rats. The concentration and integrity of the isolated RNA samples was determined using Nanodrop1000 (NanoDrop Technologies, Thermo Fischer Scientific, Waltham, MA, USA) and Agilent Bioanalyzer RNA kit. Samples were then provided to the University of Chicago Genomics Core Facility (RRID: SCR_019196) for RNA sequencing. cDNA library construction was carried out with 100 ng of total RNA input followed by 100 bp paired-end sequencing (using PolyA+ selection) on an Illumina NovaSEQ 6000 platform.

### 2.3. mRNA-Seq Bioinformatics Analysis

The processing pipeline of the sequencing data by the University of Chicago’s CRI Bioinformatics core is described as detailed below. The quality of raw sequencing reads was conducted with Fast QC and pre-processed to trim low-quality reads and remove adapters. The reads were mapped to the rat reference transcriptome (Rnor_6.0.102) using STAR version 2.6.1d, which was used to summarize data from transcript level to gene level (Ensembl 84 reference genomes). Following read alignment, the gene expression profiles were quantified using Salmon to obtain the Fragments Per Kilobase of transcript per million mapped reads (FPKM). The matrix of read counts and the design file were imported to R and, subsequently, voom normalized before being subjected to downstream descriptive statistics analysis. To identify differentially expressed genes, data were normalized using the TMM method in the Edge R package. The raw RNA-Seq data are accessible via GEO (GSE #252955). Volcano plots were created using ggplot2 R package.

### 2.4. Functional Annotation Analyses with Identified DEGs

To obtain a complete picture of gene expression changes associated with development, we performed ranked gene list analyses using gene-set enrichment analysis (GSEA) software (v4.2.2) [18]. In the GSEA method, transcript abundance was analyzed from a ranked list of all available genes to identify the regulation of functionally related gene sets with statistically significant enrichment. Analyses in GSEA were performed on normalized counts generated in Edge R and for annotated genes only. Functional enrichment of DEGs was performed by the gene ontology biological process (GOBP), Kyoto encyclopedia of genes and genomes (KEGG), REACTOME, BIOCARTA, and HALLMARK databases. An absolute fold change ≥ 2 and false discovery rate (FDR) adjusted *p*-value threshold was set to *p* < 0.05 for up- and downregulated genes.

### 2.5. Protein–Protein Interactions between DEGs

PP1 (protein–protein interaction) network of DEGs was constructed using the STRING database. A protein interaction map was uploaded to Cytoscape (http://apps.cytoscape.org/, accessed on 17 November, 2022) to find core node clusters. Cytohubba application in Cytoscape was applied to select the top 10 most central or key hub nodes ranked by the MCC (maximal clique centrality) algorithm.

### 2.6. QRT-PCR (Quantitative Reverse Transcription-PCR)

QRT-PCR was performed using an iScript cDNA synthesis kit and SsoFast EvaGreen (Bio-Rad, Hercules, CA, USA) as a fluorogenic binding dye. Housekeeping genes 18S RNA and β-actin were included as controls for quantitation of data. Changes in target mRNA expression were calculated based on the Δ(ΔCt) method [19], and data are shown as mean ± SEM. Statistical significance defined by *p* ≤ 0.05 was determined by one-way ANOVA followed by a post hoc Tukey HSD. Primer sequences used in this study are listed in Supplementary Table S1.

## 3. Results

### 3.1. Generating RNA-Seq Data for Differential Expression Analysis from Neonatal and Adult CBs

**Figure 1:**
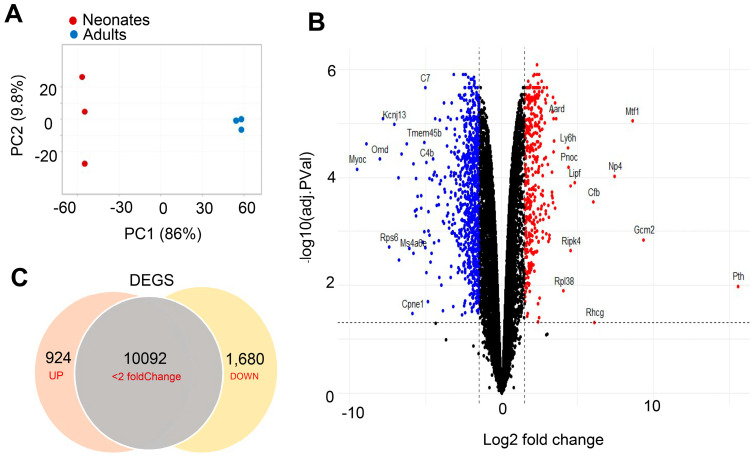
Transcriptional patterns and changes in gene expression analysis in neonatal (neo) and adult (adu) CBs. RNA sequencing was performed from RNA isolated from 3 biological replicates. For each biological replicate, CBs from 8 pups or 4 adults were pooled together. (**A**) Principal component analysis of gene expression patterns for CBs from neonates (red dots) and adults (blue dots). (**B**) Volcano plot showing –log10 (adj *p* values) as a function of log2 (fold change) between neonates and adults. The log10 (adj *p* values) represents the level of significance of each gene, while log2 (fold change) represents the difference between the levels of expression for each gene, with colored dots representing significantly differentially expressed genes. The significantly overexpressed genes are indicated in red, whereas significantly under-represented genes are indicated in blue, along with the names of the most regulated genes highlighted next to the dots. (**C**) Venn diagram illustrating differentially expressed genes (absolute fold change ≥ 2 and *p*-value ≤ 0.05). A total of 924 genes were upregulated, 1680 genes were downregulated, and 10,092 showed no change.

RNA-seq analysis was performed on CBs harvested from neonate (day 5) and adult (day 21) SD rats. Day 5 and day 21 rats were chosen because (1) CB hypoxic chemosensitivity increases during the first 2–4 weeks of postnatal development. and (2) gestational and the weaning age for rats is around 21 days. RNA sequencing was performed from RNA isolated from three biological replicate samples (*n* = 3). For each biological replicate, CBs from eight pups or four adult rats were pooled to obtain sufficient material for RNA sequencing. The data obtained included both females and males. Hence, sex difference expressions were not taken into account in the analysis. RNA-Seq libraries were constructed with total RNA using poly A+ selection, combined with the Illumina NovasSEQ platform, which allowed us to detect most polyadenylated transcripts. The total number of reads varied between 15 and 20 million per sample. The reads were mapped to rat transcriptome (Rnor_6.0.102). Filtering was carried out to remove non-coding protein genes, which reduced the number of genes from 32,883 to 22,245. Further filtering for low expression genes (with less than 1 count per million, CPM) in both of the duplicate samples reduced the number to 13,488 genes, which were considered for differential expression analysis. Principle component analysis showed a discrete separation between neonatal and adult CB expression profiles (Figure 1A).

### 3.2. Transcriptional Regulation Differences between Neonatal and Adult Carotid Bodies

The total number of genes expressed between the two developmental stages of neonates vs. adults did not differ significantly. For example, 12,696 genes were commonly shared between both developmental stages, while very few genes (<0.25%) were found to be uniquely expressed in neonatal and adult CBs. However, comparative gene expression analysis of the RNA-Seq data identified significant changes in gene expression between neonates and adults. The distribution of differentially expressed genes (DEGs) is shown in the volcano plot mapped by statistical differential gene expression data (adjusted *p*-value) versus magnitude of expression change (Log 2-fold change) between neonates and adults, with the gene names of the most significant and highest expression change listed (Figure 1B). Of the 12,696 genes commonly shared genes, a total of 2604 genes showed differential expression by comparing the neonates with that of adults as analyzed by *t*-test (*p*-value ≤ 0.05, fold change FC ≥ 2 or ≤0.5). Of the 2604 DEGs, as many as 924 were upregulated, and 1680 genes were downregulated in neonates (Figure 1C).

### 3.3. Transcriptional Signatures and DEG Responses Associated with Neonatal CBs

**Figure 2:**
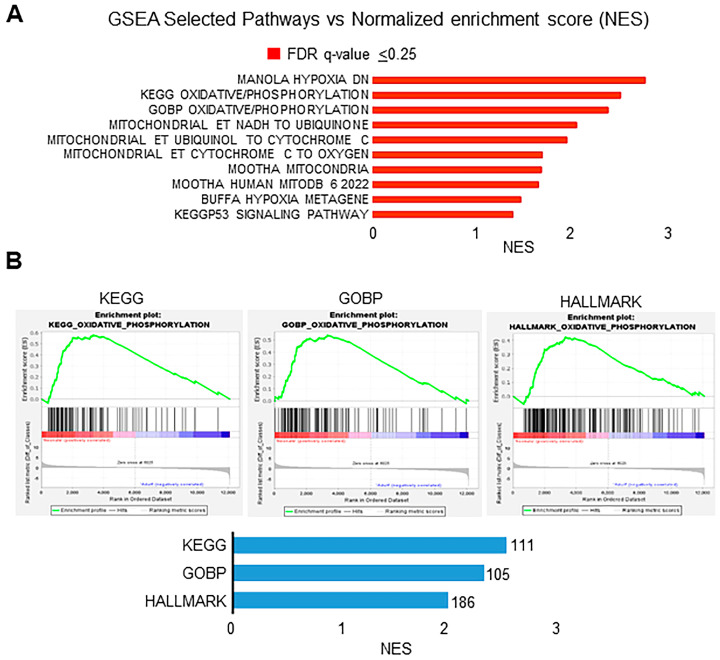
Analysis of differentially expressed genes (DEGs) in neonates compared to adults. (**A**) Gene-set enrichment analysis (GSEA) results showing enriched gene sets in neonates with normalized enrichment score (NES). Bars in red indicate significant enrichment at a false discovery rate of (FDR) Q value ≤ 0.25. (**B**) Top panel: Enrichment plots for the ox/phos gene sets in KEGG, GOBP, and HALLMARK analysis, showing the profile of running ES score and positions of genes on the rank-ordered list. Bottom panel: Number of genes in the ox/phos pathway that were enriched in neonates by gene ontology biological process (GOBP) (111 genes), KEGG pathway (105 genes), and Hallmark pathway (186 genes) enrichment analysis along with normalized enrichment score (NES).

To gain further insight into the potential function of the DEGs, we performed a gene-set enrichment analysis (GSEA) analysis. GSEA of normalized counts of genes expressed in neonates compared with those expressed in adults showed that ten gene sets were significantly enriched in neonatal CBs (FDR ≤ 25%) (Figure 2A). The top highly enriched gene sets seen in the neonatal CBs were oxidative phosphorylation (ox/phos) and hypoxia pathways, categories known to play a role in oxygen sensing. Enrichment of the ox/phos gene-set by gene ontology biological process (GOBP) (111 genes) was also confirmed by KEGG (105 genes) and Hallmark pathway (186 genes) enrichment analysis (Figure 2B).

**Figure 3:**
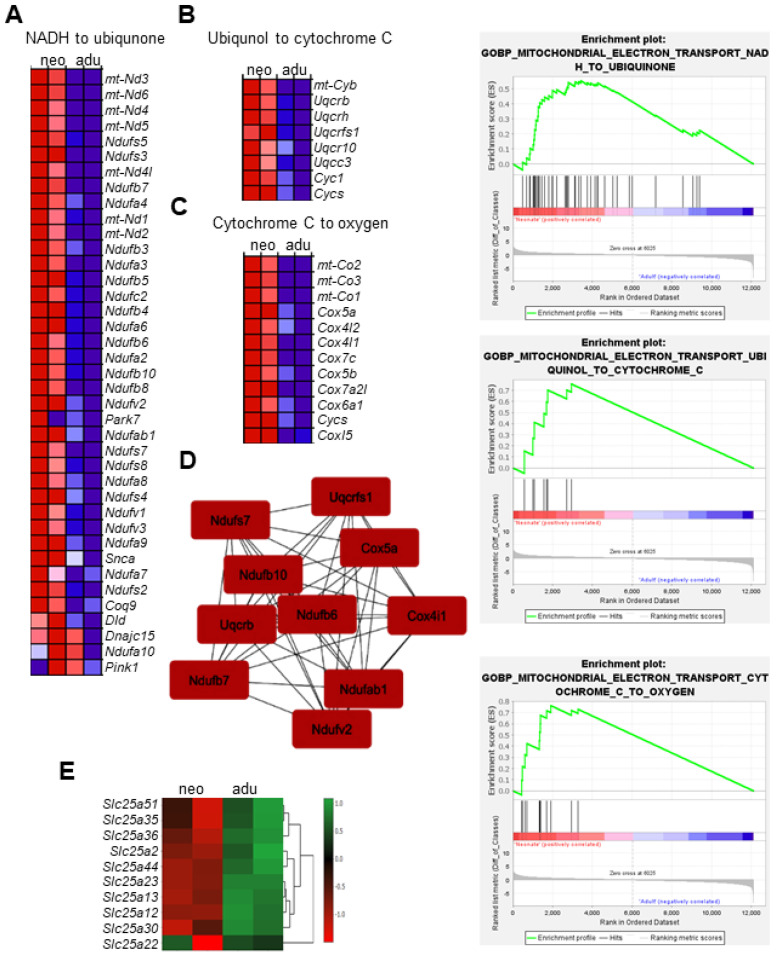
Analysis of mitochondrial ETC enriched genes in neonates. (**A**–**C**) Top panel: Heat map of the top ETC genes in comparison of neonates (left column) vs. adults (right column). Colors represent the expression values ranging from red (high expression), pink (moderate), light blue (low) to dark blue (lowest expression). Bottom panel: Enrichment plots for the mitochondrial ETC gene sets in GOBP analysis, showing the profile of running ES score and positions of genes on the rank-ordered list. (**D**) Map of interactions between proteins encoded by mitochondrial ETC genes created using protein–protein interaction analysis in STRING 11.0 (score set to medium confidence > 0.4) and visualized using the Cytohubba application in Cytoscape software. (**E**) Heat map of top significantly downregulated mitochondrial solute-carrier protein genes (Slc25) in neonates compared to adults. The scale bar represents log Fc values.

Examination of the genes within the GOBP ox/phos pathways revealed that the top 60 genes enriched in neonatal CBs are involved in the mitochondrial electron transport chain (ETC), which can be classified into three groups, shown as a hierarchical heat map in Figure 3A–C. The first group includes core subunits of complex 1 encoded by mitochondrial DNA (mtDNA): Nd1-5 as well as nuclear DNA (nDNA) *(Ndufv*s and *Nduf*s) in addition to accessory subunits (*Ndufa* and *Ndufb*), which couple H+ transport to electron transfer from NADH+ to coenzyme ubiquinone (Figure 3A). The second group consists of ubiquinol to cytochrome C reductase complex III subunits (*mt-Cytb*, *Uqcrb*, *Uqcrh*, *Uqcrfs1*, and *Uqcr10*) (Figure 3B), which generate superoxide during the transfer of electrons to cytochrome C. The third group involves complex IV or cytochrome C oxidase, the site of oxygen binding and reduction. The mitochondrial subunits (*mt Cox 1-3*), along with several *Cox* genes, were also enriched in neonates (Figure 3C).

To visualize the relationship between proteins encoded by ox/phos pathway DEG genes, protein–protein interaction (PPI) networks were constructed using the STRING database imported to Cytoscape and the MCC (maximal clique centrality) algorithm of the Cytohubba plugin. Based on MCC scores, the top 10 hub nodes in the PP1 network are shown in Figure 3D. These 10 hub nodes belonging to complexes 1, III, and IV were selected as the most central or key nodes in the network. Analysis of KEGG and Hallmark ox/phos pathway sets yielded similar results (Supplementary Figure S1).

In contrast to mitochondrial ETC genes, mitochondrial solute-carrier genes (*Slc25*) [20] related to mitochondrial function showed a decrease in neonatal CBs (Figure 3E). Most notable among them are the ATP-Mg solute carriers (*Slc25a23*) which transport adenine nucleotides to the matrix of the mitochondria in response to cytosolic Ca^2+^ [21], and Kmcp1 (kidney mitochondrial carrier protein 1, encoded by *Slc25a30*), catalyze the transport of anions and thiosulfate, which are produced by H_2_S degradation in the mitochondria and play a role in the modulation of H_2_S levels [22]. *Agc1* (*Slc25a12* or Aralar1). *Agc2* (*Slc25a13* or citrine), which is important for aspartate and glutamate [23], was also decreased in neonates. Taken together, these results suggest that genes involved in mitochondrial metabolism that have been previously shown to be associated with oxygen-sensing pathways are significantly altered with CB development.

**Figure 4:**
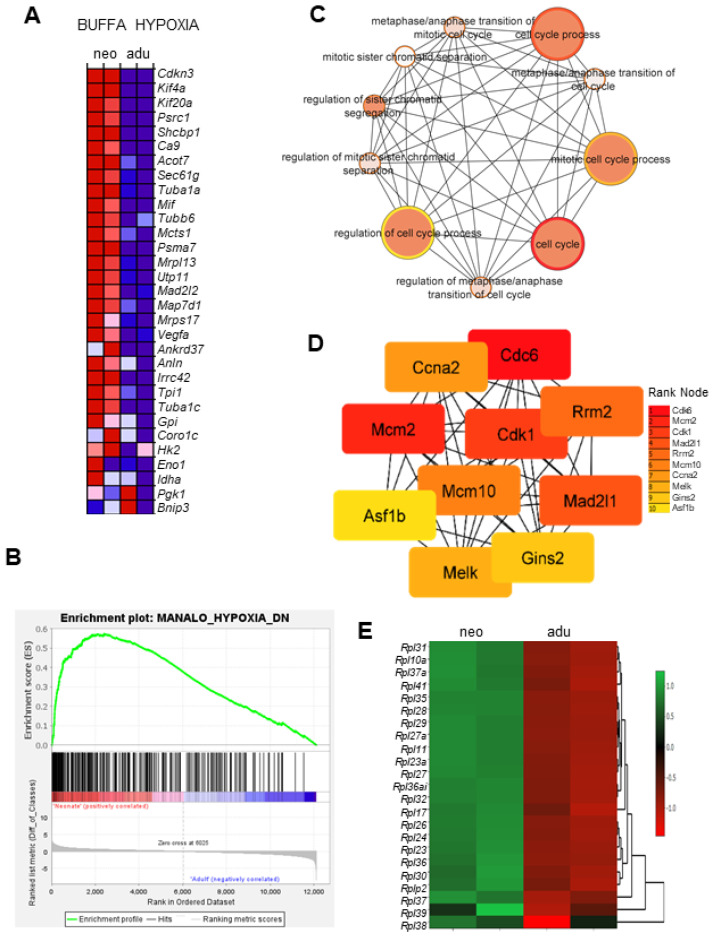
Analysis of hypoxia-regulated genes in neonates. (**A**) Heat map of the Buffa hypoxia metagene set enrichment analysis of neonates (left column) vs. adults (right column). Colors represent the expression values ranging from red (high expression), pink (moderate), light blue (low) to dark blue (lowest expression). (**B**) Enrichment plots for the Manola hypoxia-downregulated gene-set showing the profile of running ES score and positions of genes on the rank-ordered list. (**C**) Biological pathway enrichment map for the Manola hypoxia-downregulated gene-set analysis results created in Cytoscape. (**D**) Map of interactions between proteins encoded by Manola hypoxia-downregulated genes created using protein–protein interaction analysis in STRING 11.0 (score set to medium confidence and FDR < 0.05) and visualized using Cytoscape software. Colors represent the ranking of each node, ranging from red (1; high expression) to yellow (10; lowest expression). (**E**) Heat map of top significantly upregulated ribosomal proteins (Rpl) in neonates compared to adults. The scale bar represents logFc values.

Besides the ox/phos pathway, GSEA analysis revealed hypoxia as another highly enriched gene-set in the neonatal CBs (Figure 2A). Interestingly, only 32 genes upregulated by hypoxia were enriched as analyzed by the Buffa hypoxia metagene database (Figure 4A). Included among these are the known HIF-1 target genes *Vegfa* and glycolytic enzymes (*Gp1*, *Tpi1*, *Gpi*, *Hk2*, *Eno1*, and *Ldha*).

In contrast, the large category of genes (~180) known to be downregulated by hypoxia were enriched in neonates as analyzed by Manola hypoxia gene-set enrichment analysis (Figure 4B). Further analysis of the 180 genes showed that the genes were classified to regulation of cell cycle process pathways (Figure 4C). PP1 network using the MCC algorithm of the Cytohubba plugin mapped the top highly scored genes (Figure 4D) as the most central or important nodes in the network. Included among these are genes *Cdk1*, *Ccna2*, and *Cdc6*, which encode proteins involved in cell growth/proliferation. In addition, several ribosomal proteins involved in cell growth and development were also enriched in neonates (Figure 4E).

**Figure 5:**
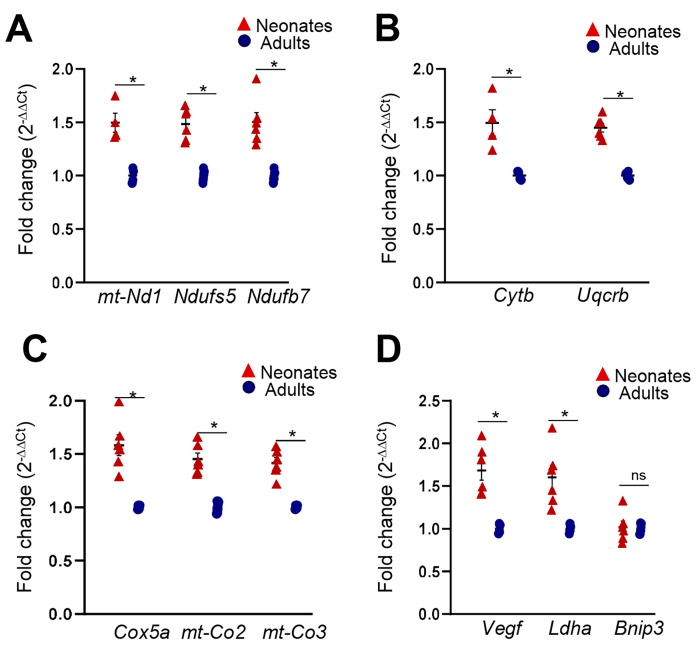
mRNA expression of (**A**) *mt-Nd1*, *Ndufs5* and *Ndufb7* subunits of complex 1, (**B**) *mt-Cytb*, and *Uqcrb* subunits of complex III, (**C**) *Cox5a*, *mt-Co2* and *mt-Co3* subunits of complex IV or cytochrome C oxidase, and (**D**) Hif-1 target genes *Vegf*, *Ldha*, and *Bnip3* in CBs of neonates and adults as analyzed by qRT-PCR. Data show as mean ± SEM, n = 5 biological replicates. * *p* values ≤ 0.05.

RNA-seq data were validated by performing real-time RT-PCR for a few selected genes from ox/phos and hypoxia pathways. The qRT-PCR confirmed that expression of the selected genes increased more than 1.5-fold in neonates compared to adults (Figure 5A–D).

### 3.4. Transcriptional Signatures and DEG Responses Associated with Adult CBs

**Figure 6:**
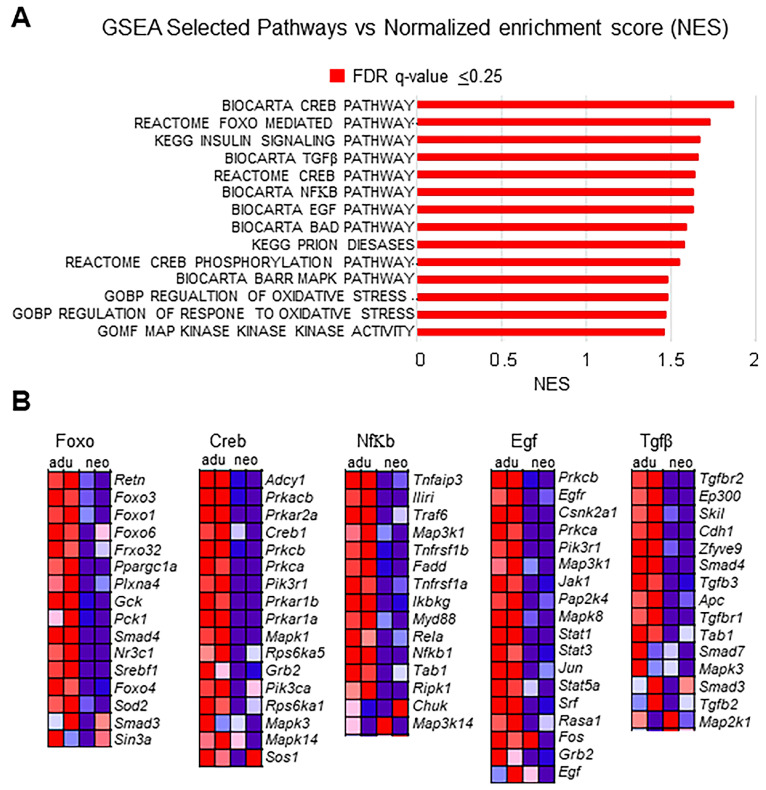
Analysis of differentially expressed genes (DEGs) in adults compared to neonates. (**A**) GSEA analysis of enriched gene sets in adults with normalized enrichment score (NES). Bars in red indicate significant enrichment at a false discovery rate of (FDR) Q value ≤ 0.25. (**B**) Heat map of the Foxo, Creb, NfΚb Egf, and Tgfβ, mediated transcription pathway gene-set enrichment analysis of adults (left column) vs. neonates (right column). Colors represent the expression values ranging from red (high expression), pink (moderate), light blue (low) to purple (lower) and dark blue (lowest expression).

GSEA of normalized counts of genes expressed in adults compared with those expressed in neonates showed that close to 14 gene sets were significantly enriched in adult CBs (FDR ≤ 25%) (Figure 6A). The top highly enriched gene sets included transcription factor (Creb, Nfkβ, and Foxo) and growth factor (Egf and Tgfβ) pathways. Examination of the genes within the Creb and Nfkβ pathways revealed that the top genes upregulated in adult CBs included cAMP-dependent protein kinases (Prkca, Prkcb, Pik3ca) and Mapk (Mapk, Mapk1, Mapk3, and Map3k1) (Figure 6B). In addition, Foxo transcription factors (sub-family of Forkhead transcription factors), Foxo1, Foxo3, Foxo4, and Foxo6, regulators of cellular quality control, were also enriched in adults (Figure 6B). Genes upregulated in adults within the EGF pathway included JAK1, STAT1, and STAT3, as well as previously mentioned Mapk (Mapk3, Mapk8, Map3k1, and Map2k4) (Figure 6B). Tgfβ2, Tgfβ3, Tgfβr1, and Tgfβr2 genes of the Tgfβ signaling pathway and Smad proteins (Smad4, Smad7, and Smad3), which are known to be phosphorylated and activated by TGFβ, were also upregulated in adults (Figure 6B). These results suggest that in adults, the expression of genes known to be associated with development is enriched.

**Figure 7:**
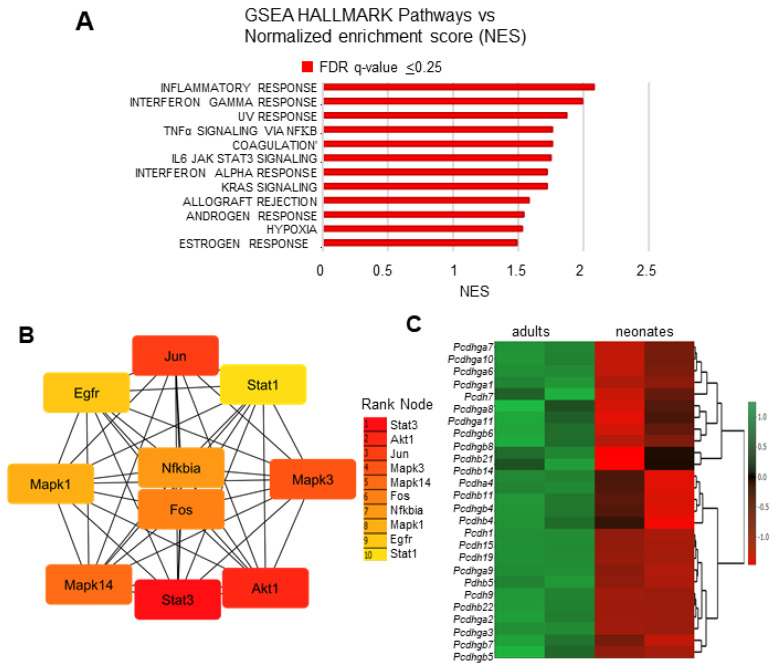
(**A**) GSEA hallmark pathways analysis of differentially expressed genes (DEGs) in adults. vs. normalized enrichment score (NES). Bars in red indicate significant enrichment at a false discovery rate of (FDR) Q value ≤ 0.25. (**B**) Combined map of protein interactions encoded by the top three gene sets (Ifnγ, Tnfα, and Il6-Jak-Stat signaling pathways) created using protein–protein interaction analysis in STRING 11.0 (score set to medium confidence and FDR < 0.05) and visualized using Cytoscape software. Colors represent the ranking of each node, ranging from red (1; high expression) to yellow (10; lowest expression). (**C**) Heat map of top significantly upregulated procadherin (*Pcdh*) genes in adults compared to neonates. The scale bar represents logFc values.

Analysis based on the Hallmark database identified cytokine signaling pathways such as Ifnγ, Tnf-α (Tumor necrosis factor), and Il6 as the top highly enriched gene sets, followed by androgens and estrogens response pathways known to be critical regulators of mammalian physiology and development (Figure 7A). Combined analysis of the Ifnγ, Tnfα, and Il-6 signaling responses by the PPI network identified proteins corresponding to genes such as *Stat1*, *Akt1*, *Jun*, *Mapk3*, *Mapk14*, *Fos*, *Nfkbia, Mapk1*, *Egfr*, and *Stat1* as the central most important nodes enriched in adults (Figure 7B), some of which are also activated by ligands such as Egf. In addition, 28 procadherin genes involved in neural circuit assembly were also positively regulated in adult CBs (Figure 7C).

## 4. Discussion

Gene ontology analysis of the transcriptomic response of CBs from neonates (day 5) and adults (day 21) identified several processes and functions that were differentially regulated with development. Based on GSEA enrichment analysis, we were able to identify significant increases in genes associated with oxidative phosphorylation in neonatal CBs compared to adults. As well as an apparent increase in the ETC subunits, analysis of the hypoxia-signaling pathways revealed an increase in the Hif-1-activated glycolytic pathways in neonatal CBs. These results suggest that increased mitochondrial activity, as well as increased hypoxia-activated glycolytic proteins resulting in high ATP levels, may be associated with the high metabolic energy demands in neonates compared to adults, which in turn may contribute to the phenotype of neonatal CB hypoxic sensitivity.

Mitochondria, which generate cellular energy in the form of ATP via oxidative phosphorylation (ox/phos) pathways, play a central role in redox metabolism and calcium homeostasis. Mitochondria have long been linked to acute CB oxygen sensing either due to the sensitivity of glomus cells to ETC inhibitors [9] or due to the presence of unusual cytochromes with reduced oxygen affinities [24,25]. Transcriptomic analysis of the whole CB showed that expression of close to 60 Complex I, III, and IV subunits of the electron transport chain (ETC) genes, which included both catalytic and non-catalytic subunits, were altered in neonates. More recent studies have shown the involvement of mitochondrial subunits Ndufs2 in CB hypoxic sensing [26]. However, our study did not show any significant change in the expression of the *Ndufs2* gene with development. Instead, *Ndufs7*, *Ndufb6,* and *Ndufb7* subunits of complex I were identified as a part of the central most important nodes.

Single-cell transcriptomic profiling of CB glomus cells identified two atypical mitochondrial subunits (*Ndufa412* and *Cox4i2*) in glomus cells, both of which are induced under long-term and severe hypoxia [17]. Cox4i2 (the isoform 2 of the subunit 4 of the cytochrome oxidase) protein was shown to be an essential component in the oxygen-sensing process of the pulmonary vasculature by increasing Cox activity and promoting hyperpolarization of mitochondria resulting in reactive oxygen species (ROS) production [27]. Interestingly, both *Cox4i1* and *Cox4i2* genes not only showed high expression in whole CB transcriptomic analysis but also were enriched in neonates. This raises an interesting question of whether mitochondria of non-glomerular cells like endothelial cells in the CB, which is a highly vascularized tissue, also express these subunits similar to glomus cells and, if so, their contribution to hypoxic sensing.

Mitochondrial solute-carrier proteins (Slc25 members) found in the inner mitochondrial membrane facilitate the transport of molecules involved in the urea and citric acid cycles, oxidative phosphorylation, DNA maintenance, and iron metabolism, among other processes [28]. Surprisingly, our analysis identified a few of the Slc25 members, specifically those important for the transport of adenine nucleotides, thiosulfates, aspartate, and glutamate, which were significantly decreased in neonates. Ox/phos homeostasis is tightly controlled by mitochondrial transporters located in both the inner and outer mitochondrial membrane. Further studies are needed to understand if there is any causal relationship between the decreased expression of Slc25 members and the increase in the ETC subunits, as well as their role in hypoxic sensing.

One of the mechanisms proposed for the low hypoxic sensitivity of neonatal CB is the low PaO_2_ of the pre-natal environment vs. relatively O_2_-rich postnatal environment, raising the possibility that hypoxia-related adaptations in neonates may be lost with development. HIF-1α is a transcriptional activator that functions as a global regulator of O_2_ homeostasis, modulating embryonic development as well as a variety of postnatal physiological adaptations to hypoxia [29,30]. Although our results indicated that only a few Hif-1-regulated target genes like Vegfa and related glycolytic enzymes were upregulated in neonates, a greater number of genes that were normally repressed by hypoxia were enriched in neonates. These genes mostly encode proteins involved in cell growth/proliferation. We speculate that these genes may play a role more in development than contributing to the hypoxic sensitivity of CB.

It is noteworthy that neonatal transcriptome changes are consistent with the hypoxic environment in the neonatal period. It would be interesting to see in future studies whether adult rats subjected to 21 days of hypoxia (equivalent to gestational duration) mimic transcriptome profiles of neonatal CBs. Such an analysis is physiologically relevant because prolonged hypoxia attenuates CB sensitivity to acute hypoxia [31], similar to attenuated CB hypoxic sensitivity in neonates

In our analysis, we found that the JAK-STAT pathway was enriched in adults compared to neonates. This pathway is known to be regulated by a wide range of cytokines like Tnfα as well as growth factors. Importantly, recent studies have shown that CB is a key player in neuro-immune interactions [32,33], and the presence of functional Tnf-α and interleukins (Il-1β, Il-6) receptors that activate CB have been reported [34,35]. Whether the upregulation of the cytokine signaling pathway represents a more generalized pattern of age-dependent development of functional immune competence or contributes to heightened CB hypoxic sensitivity in adults needs further investigation.

Balbir et al. examined the differential gene expression in the CBS of two strains of mice (DBA/2J and A/J) and showed that variable expression of potassium channels, neurotransmitters, and neuromodulator genes may be associated with differences in CB hypoxic sensitivity between the two mice strains [15]. Comparison of our analysis with the published data showed significant changes in the reported genes, especially *Gdnf*, *Kcnmb2*, *Kcnq2*, *Phox2b*, *Nos1*, *Gfap*, *Bmf*, and *Arnt2* with development, suggesting a potential role for neuromodulators in CB hypoxic sensitivity in addition to their importance across species (rats vs. mice). Single-cell transcriptomic analysis of mouse carotid body glomus cells identified G-protein-coupled receptor (Olfr78) protein implicated in CB function as one of the most abundant genes [36]. Interestingly, our analysis showed that *Olfr59* (the rat equivalent to mouse *Olfr78*) gene was the only abundant Olfr member identified in the rat CB transcriptomic profile, highlighting the significance of G-protein signaling in different species. Peng et al. [37] recently showed that redox modification of Olfr78 by H_2_S contributes to CB hypoxic sensing. In our study, *Olfr59* gene expression increased significantly (> 2-fold) in adult CBs compared to neonates. Future studies are required to determine whether Olfr59 plays a causal role in postnatal CB hypoxic sensitivity.

In conclusion, at the genomic level, postnatal maturation of the CB appears to involve several pathways, including hypoxia, cytokine, and G-protein signaling, in addition to the well-studied mitochondrial pathways. Since glomus cells are critical for CB hypoxic sensing, it is conceivable that all the genetic differences observed with whole CB in this study could be attributed in part to changes in glomus cells. However, further investigation of these pathways under hypoxic conditions, specifically in different cell populations of CB, followed by loss of function studies, is required to understand the molecular mechanisms that give rise to postnatal CB hypoxic sensitivity. Finally, knowledge of genetic factors regulating CB chemosensitivity in perinatal development will provide a useful resource to address other aspects related to diseases involving CB, like sleep apnea and sudden infant death syndrome.

## Data Availability

RNA-seq data generated for the current study are available in the NCBI Gene expression omnibus (GEO) repository, accession number GSE 252955.

## Supplementary Materials

The following supporting information can be downloaded at: https://www.mdpi.com/article/10.3390/genes15030302/s1, Table S1: Primer sequences for QRT-PCR amplification. Figure S1: Protein protein interaction analysis of ox/phos gene sets obtained by (A) KEGG and (B) HALLMARK enrichment analysis.
