# Supplementary material for: Transcriptomic Analysis of Postnatal Rat Carotid Body Development
Source: Genes (Basel). 2024 Feb 27;15(3):302. doi: 10.3390/genes15030302 (PMC10970570; doi:10.3390/genes15030302)
Supplement: Supplementary file 1 [file genes-15-00302-s001.zip › genes-2886155-supplementary.pdf]

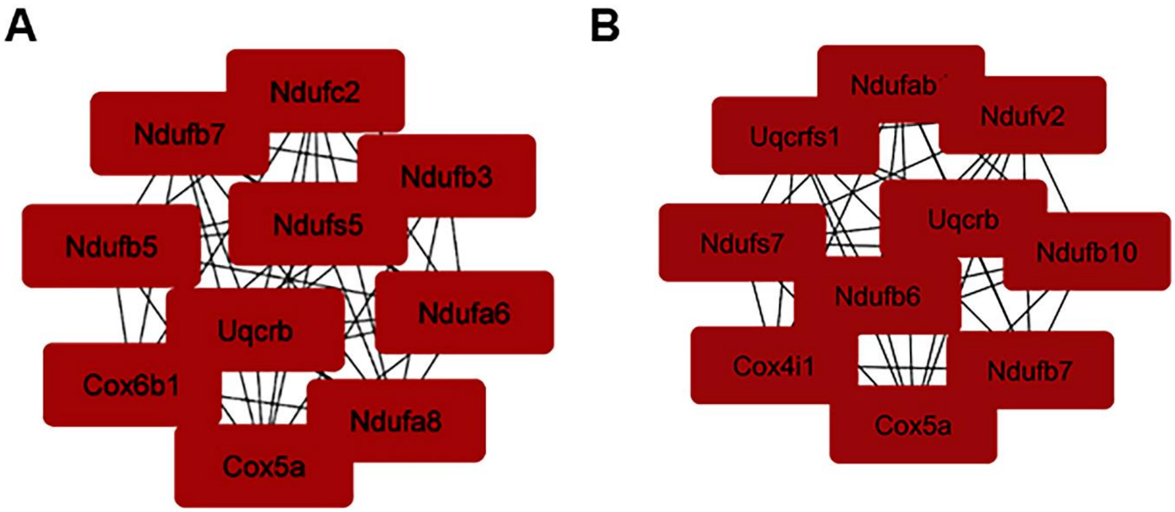

Figure S1: Protein protein interaction analysis of Ox/phos gene sets obtained by A) KEGG and B) HALLMARK enrichment analysis. The top 10 hub nodes encoded by mitochondrial ETC genes created using protein–protein interaction (PPI) analysis in STRING 11.0 (score set to medium confidence > 0.4) and visualized using the Cytohubba application in Cytoscape software

Table S1: Primer sequences for QRT-PCR amplification

Gene	NCBI ref	Forward and reverse primer sequences	Amplicon length(bp)
Mt ND1	NC_001665.2	Fwd: TGG TAT TCT ACA ACC ATT TG<br>Rev: GGG GGT GAG GTA TTG GTA AG	155
Ndufb7	NM_001108442	Fwd: CTGAAGTGCAAACGCGACAG<br>Rev: CCGCTCACGTTCAAACTCCT	120
Ndufs5	NM_001030052.1	Fwd: CAT TGC TGA GGT GCG GTA GG<br>Rev: AGC GGC ATT CTT GTA AGG CT	143
CytB	NC_001665.2	Fwd: CCC GCC CCA TCT AAC ATC TC<br>Rev: CCG TAG TTT ACG TCT CGG CA	164
Uqcrb	NM_001127553.2	Fwd: TGC AGC GGG CTT CAATAA AC<br>Rev: GGC AGC CTT CTT ATG GCT TCT	87
Cox5a	NM_145783.1	Fwd: ATG CTC GCT GGG TGA CAT AC<br>Rev: AGA TGC GAA CAG CAC TAG CA	168
Mt CO2	NC_001665.2	Fwd: TGG CTT ACA AGA CGC CAC AT<br>Rev: TGG GCG TCT ATT GTG CTT GT	156
Mt CO3	NC_001665.2	Fwd: CCA CCA AAC CCA TGC ATA CC<br>Rev: GGT GGC CTT GGT ATG TTC CT	206
VEGF	NM_031836.3	Fwd: AAT GAT GAA GCC CTG GAG TG<br>Rev: TAT GTG CTG GCT TTG GTG AG	90
LDHA	NM_017025.1	Fwd: CAA GAG GGA GAG AGC CGG<br>Rev: TCC AAG CCA CGT AGG TCA AG	145
BNIP3	NM_053420.3	Fwd: TCC TGG GTA GAA CTG CAC TTC<br>Rev: GCT GCG CAT CCA GCA GTA TTT	103
18s	NR_046237.1	Fwd: CGC CGC TAG AGG TGA AAT TC<br>Rev: CGA ACC TCC GAC TTT CGT TCT	101
